# Prior Appendectomy and the Onset and Course of Crohn's Disease in Chinese Patients

**DOI:** 10.1155/2019/8463926

**Published:** 2019-07-17

**Authors:** Dafan Chen, Jing Ma, Qiwen Ben, Lungen Lu, Xinjian Wan

**Affiliations:** ^1^Department of Gastroenterology, Shanghai General Hospital, Shanghai Jiao Tong University School of Medicine, Shanghai 200080, China; ^2^Department of Gastroenterology, Ruijin Hospital, Shanghai Jiao Tong University School of Medicine, 197 Ruijin Second Road, Shanghai 200025, China

## Abstract

**Background and Aim:**

The relationship between prior appendectomy and Crohn's disease (CD) has previously revealed conflicting findings. The present study investigates the relationship between prior appendectomy and CD development in Chinese patients.

**Methods:**

A retrospective case-control study was performed to compare prior appendectomy rate between CD patients and age- and gender-matched controls at two Chinese hospitals. The clinical course of CD was determined in patients who underwent and did not undergo appendectomies before CD diagnosis.

**Results:**

A total of 617 CD patients and 617 controls were included. The appendectomy rate before CD diagnosis in patients was higher, when compared to controls (6.65% versus 3.73%, *P* = 0.033). Appendectomy was a risk factor for the onset of CD independent of smoking in the multivariate analysis (OR: 1.878; 95% CI: 1.111–3.174; *P* = 0.019). Appendectomies were performed closer to the date of CD diagnosis in the trend test (*P* = 0.039). The rate of appendectomy within one year or 1-5 years before CD diagnosis was higher in patients when compared to that in controls (0.97% versus 0%, *P* = 0.031; 1.13% versus 0.32%, *P* = 0.180). However, the rate of appendectomy over five years before CD diagnosis was close to controls (4.54% versus 3.40%, *P* = 0.392). No significant differences in disease location, behavior, medication, and intestinal resection between appendectomy and nonappendectomy CD patients were found, even in the subgroup analysis by age of appendectomy.

**Conclusion:**

Prior appendectomy is a risk factor for the onset of CD. However, the appendectomy rate only increased for a short duration before CD diagnosis, likely reflecting a diagnostic bias. Prior appendectomy did not influence the features or course of CD.

## 1. Introduction

Inflammatory bowel diseases (IBD), including Crohn's disease (CD) and ulcerative colitis (UC), are chronic inflammatory disorders of the gastrointestinal tract. IBD is considered as a multifactorial disease that involves the interaction between genetic and environmental factors, which give rise to an abnormal immunologic response [1, 2]. The investigation of risk factors may help to better understand the etiology and pathogenesis of IBD.

The appendix is an immune organ that supports the immunity process. Appendectomy could modulate the gut immune function, thereby influencing the occurrence of intestinal inflammation [3–5]. Many studies have reported the inverse relationship between appendectomy and the incidence of UC [6–11]. Previous studies have demonstrated that the effects of appendectomy in CD are inconsistent and less certain. However, a few study results have revealed that the risk of CD increases after appendectomy [12–17], while a few other studies revealed a decreased risk [8, 18] or insignificant changes [10, 11, 19–22]. Appendectomy, whether included or not, and when performed close to the time of CD diagnosis may influence the statistical results. A meta-analysis study revealed the elevated risk early after an appendectomy but diminishes thereafter [23]. In a cohort study conducted in Sweden and Denmark, the occurrence of CD increased during the first year after appendectomy but not significantly after five years, and this likely reflects the differential diagnostic problems in CD patients [17]. However, a cohort study conducted in Sweden revealed that the risk of CD remained high even in cases at 20 years after appendectomy [14]. In addition, data on the effects of appendectomy on the clinical course of CD are less and inconsistent. Two studies suggested that appendectomy before CD diagnosis was associated with worsened clinical course in patients [14, 24]. Another two studies suggested that appendectomy performed before CD diagnosis did not affect the severity of the disease [25]. Furthermore, the clinical characteristics of CD varies among different ethnic groups [26, 27]. Data regarding the effect of appendectomy on CD onset and its clinical course in Chinese patients are scarce. According to a study, prior appendectomy increased the risk of CD development in Chinese patients, but the number of enrolled patients was small (merely 102 CD patients), and this study lacked an in-depth analysis [28].

Hence, the present study aimed to investigate the relationship between appendectomy performed before CD diagnosis and CD onset and discussed its clinical course in the Chinese population.

## 2. Methods

### 2.1. Study Design

A retrospective study was conducted at the Department of Gastroenterology in two hospitals in Shanghai, China. The Investigation and Ethics Committee of Shanghai Jiao Tong University School of Medicine approved the present study.

A retrospective case-control study was performed to compare the incidence of appendectomy between CD patients and controls. The relevant data was obtained from clinical medical charts. Patients were contacted through a questionnaire or by telephone when the detailed information was unclear. Consecutive CD inpatients who attended these two hospitals between January 2013 and December 2017 were included. CD was diagnosed according to the clinical, endoscopic, histological, and/or radiologic criteria [29]. Patients who underwent an operation of pure appendectomy prior to the diagnosis of CD without intestinal resection were considered in the appendectomy group. Patients were excluded when specific information regarding the appendectomy or CD was lacking, such as whether an appendectomy was performed, whether the appendectomy was performed with intestinal resection or others, and whether the appendectomy was performed before the diagnosis of CD. The time of appendectomy and diagnosis of CD were carefully assessed. Patients in the control group included age-matched (±2 years old) and gender-matched patients, who visited these two hospitals during the same period, and had minimal gastrointestinal diseases, such as established dyspepsia, gastrointestinal polyp (<0.5 cm in diameter), *Helicobacter pylori*- (Hp-) related peptic ulcer, reflux esophagitis, and mild acute infectious gastroenteritis. At present, these minimal gastrointestinal diseases are not considered to be correlated to appendectomy. Clinical controls could reduce the population selection bias compared to community controls in the previous Chinese study [28]. Patients with IBD or suspected of IBD, or patients who lacked specific information regarding the appendectomy, were excluded from the control group. The matching ratio was 1 : 1.

The age at CD diagnosis, location of the CD, disease behavior, the use of drugs, and the intestinal resection were compared between CD patients who underwent and those who did not undergo appendectomies before CD diagnosis. The results were used to determine whether the appendectomy before CD diagnosis affected the phenotype and course of CD.

Demographic and clinical data were retrieved, which included gender; date of birth; nationality (classified as Han nationality and minority); date of CD diagnosis; disease duration; history of appendectomy; smoking status (classified as nonsmoker if they never or rarely smoked and smoker); alcohol drinking (classified as nondrinker if they never or rarely drinking and drinker); use of nonsteroidal anti-inflammatory drugs (NSAIDs; classified as never used and used); disease location (maximum extent recorded); medication use (steroids, immunosuppressants [azathioprine, 6-mercaptopurine, or methotrexate], and anti-TNF antibodies); and surgical treatment history. These CD patients were classified using the Montreal classification [30].

### 2.2. Statistical Analysis

Continuous data were expressed as mean ± standard deviation or median with range. The differences between the groups were analyzed by Student's *t*-test for normally distributed data or Wilcoxon rank sum test for abnormally distributed data. Category variables were compared by chi-square test or Fisher's exact test. The trend test was used to compare the intervals between appendectomy and CD diagnosis. In the univariate analysis, variables with *P* value of <0.10 entered the multivariate analysis. The multivariate analysis was performed through the logistic regression analysis. A two-tailed *P* value of <0.05 was considered statistically significant. All statistical analyses were conducted using SPSS version 19.0 (SPSS, Chicago, IL, USA).

## 3. Results

### 3.1. Clinical Characteristics of Patients and Controls

During the study period, a total of 621 CD inpatients visited the two hospitals. Among these patients, four CD patients were excluded due to lack of specific information regarding the appendectomy or CD. In the excluded CD patients, two patients were not sure about whether an appendectomy was performed, while the other two patients did not know whether an appendectomy was performed with intestinal resection and were not sure about the date of CD diagnosis. Finally, 617 CD patients were enrolled in the present study. In addition, 617 age- and gender-matched subjects, who visited these hospitals, were included as controls. Approximately 35% of the controls had dyspepsia, 20% had Hp-related peptic ulcer, 15% had gastrointestinal polyps, 15% had reflux esophagitis, and 15% had mild acute infectious gastroenteritis. Approximately one-third of the included CD patients and controls were from outside of Shanghai city. The clinical characteristics of CD patients and controls are presented in Tables 1 and 2.

### 3.2. Comparison of CD Patients with Controls

The rate of appendectomy performed prior to CD diagnosis in patients was significantly higher when compared to that of in the controls (41, 6.65% versus 23, 3.73%, *P* = 0.033). None of the patients underwent an appendectomy without intestinal resection after CD diagnosis. In order to investigate the relationship between appendectomy and the phenotype of CD, a subgroup analysis was performed on the basis of disease localization and the behavior of CD patients according to the Montreal classification. The analysis per stratum either in the localization or in the behavior classification in CD patients revealed no significant differences in appendectomy rate, when compared to controls (Table 3). However, a trend of increased appendectomy rate was found in terminal ileum diseases in the subgroup analysis (Table 3).

The age distribution of appendectomy, the interval between the appendectomy and CD diagnosis, and the interval between the appendectomy of a control and time of CD diagnosis of the matched patient are presented in Table 4. The appendectomy was performed at a median age of 21 years old (range: 3-48 years old) in the CD group, which was similar to controls (median age: 21 years old; age range: 4-45 years old; *P* = 0.574). When comparing the interval between the appendectomy and CD diagnosis and interval between the appendectomy of a control and time of CD diagnosis of the matched patient, the appendectomy was performed significantly closer to the date of CD diagnosis in the trend test (*P* = 0.039). The appendectomy rate within one year prior to the diagnosis of CD was significantly higher in CD patients, when compared to controls (0.97% versus 0%, *P* = 0.031), and the appendectomy rate within 1-5 years before the diagnosis of CD was also higher in CD patients, but the difference was not statistically significant (1.13% versus 0.32%, *P* = 0.180). If merely the interval between the appendectomy and diagnosis of CD over five years was considered, the appendectomy rate of the CD group was close to the controls (4.54% versus 3.40%, *P* = 0.392). When appendectomy patients were divided into subgroups by age of appendectomy of 20 years in the present study, the results revealed no significant differences between the CD group and controls in appendectomy rate (≤20 years, *P* = 0.229; >20 years, *P* = 0.099).

Several factors may affect the occurrence of intestinal inflammation.  Table 1 shows that the percentage of smokers in the CD group was significantly higher than that controls. The multivariate analysis of appendectomy and smoking demonstrated appendectomy as a significant risk factor for the onset of CD independent of smoking status (OR: 1.878; 95% CI: 1.111-3.174; *P* = 0.019), and smoking was also as an independent risk factor for CD (OR: 1.370; 95% CI: 1.077-1.742; *P* = 0.010). This effect of appendectomy on CD development was also significant when only patients who underwent an appendectomy within five years before CD diagnosis were considered (OR: 7.017; 95% CI: 1.574-31.286; *P* = 0.011). However, the multivariate analysis of patients who were appendectomized before CD diagnosis over five years did not consider appendectomy as a significant risk factor (OR: 1.367; 95% CI: 0.766-2.438; *P* = 0.290). Smoking remained as a significant risk factor for CD and was not significantly affected by the interval between appendectomy and CD diagnosis.

### 3.3. Comparison of CD Patients Who Underwent and Did Not Undergo an Appendectomy before CD Diagnosis

The relationship between the appendectomy performed before CD diagnosis and the clinical course of CD patients was examined. The clinical course from the date of CD diagnosis was retrospectively analyzed. The disease course was assessed by age at diagnosis, extent of disease, disease behavior, need for immunosuppressive therapy, and intestinal resection. CD patients were divided into appendectomy group, patients who underwent an appendectomy before CD diagnosis, and no appendectomy group, patients who did not undergo an appendectomy before CD diagnosis. The appendectomy and no appendectomy groups demonstrated similar time periods of disease course (53.24 ± 41.81 versus 47.93 ± 42.71 months, *P* = 0.441). The age at CD diagnosis in the appendectomy group revealed no significant differences, when compared to the no appendectomy group (median and range of age: 33, 15-70 years old versus 29, 6-79 years old; *P* = 0.372). During the disease course period, there were no significant differences in location of the disease, disease behavior, need for medication use, and intestinal resection between the appendectomy and no appendectomy groups. When appendectomy patients were divided into subgroups by age of appendectomy in 20 years, it was found that age at CD diagnosis in the subgroup (age of appendectomy of >20 years) was significantly higher than that in the no appendectomy group (median and range of age: 41, 24-70 years old versus 29, 6–79 years old; *P* = 0.011), while the rate of application of immunosuppressants in the subgroup (age of appendectomy of ≤20 years) was less than that in the no appendectomy group (*P* = 0.031). Since the age of CD diagnosis would affect the doctor's selection of immunosuppressants for the adverse effects, a multivariate analysis, which included appendectomy and age of CD diagnosis, was performed to analyze the effect on immunosuppressants application. The results revealed that appendectomy was not an independent risk factor for the application of immunosuppressants in the logistic regression analysis (OR: 1.676; 95% CI: 0.821–3.421; *P* = 0.156), but age of CD diagnosis was correlated to the application of immunosuppressants (OR: 1.014; 95% CI: 1.002–1.026; *P* = 0.024). There were no significant differences in the other indexes between the appendectomy group and no appendectomy group, in the subgroup analysis by age of appendectomy in 20 years. A detailed comparison of the clinical course between the appendectomy group and no appendectomy group is presented in Table 5.

## 4. Discussion

Appendectomy may influence intestinal immune function and is correlated to intestinal inflammation [3–5, 31]. The clinical characteristics of CD varies greatly among different races [26, 27]. Most previous studies on the relationship between appendectomy and CD were performed on Caucasians. Hence, it is necessary to investigate this issue in Chinese patients due to the lack of relevant studies. According to our understanding, this is the first intensive study that investigated the effect of prior appendectomy on the onset and clinical course of CD in Chinese patients.

A retrospective case-control study was conducted to analyze the relationship between appendectomy prior to CD diagnosis and the occurrence of CD. It was found that the frequency of appendectomies performed prior to CD diagnosis was significantly higher, when compared to the control group. Next, a comparison was performed between CD patients and controls in several other factors that may affect the occurrence of intestinal inflammation. The results revealed that smoking rate in the CD group was significantly higher than that in the controls. Furthermore, substantial evidence indicated that cigarette smoking increased the risk and worsened the clinical course of CD [32]. In the present study, a regression analysis was used to control the effect of smoking, while analyzing the relationship between appendectomy and the onset of CD. The results revealed that appendectomy was a significantly risk factor for the onset of CD independent of smoking.

Meanwhile, these present results revealed that appendectomy was performed at a significantly closer time of CD diagnosis. The rate of appendectomy within one year before CD diagnosis was significantly higher in CD patients, and this was relatively higher in 1-5 years before CD diagnosis. However, the appendectomy rate of patients in the CD group was close to that of the controls, when only the interval between appendectomy and CD diagnosis over five years was considered. As it is known, some of the incipient CD patients that were still undiagnosed presented bowel symptoms, which may result in unnecessary appendectomies. The appendectomy was performed close to the time of CD diagnosis, and the appendectomy rate increased within a short time before CD diagnosis, which likely reflects a diagnostic bias. This analysis was consistent with previous reports [10, 17, 23]. A trend of increased appendectomy rate was found in terminal ileum diseases in the subgroup analysis of this study, which may reflect the diagnostic problems in incipient CD patients due to similar symptoms between terminal ileum and appendix disease. Jiang et al. in China reported that appendectomy increased the risk of development of CD in Chinese patients [28]. However, this Chinese study did not analyze the interval between appendectomy and the diagnosis of CD and did not consider the possibility that appendectomy was due to a misdiagnosed CD. In previous reports, age of appendectomy influenced the effect of appendectomy on IBD. A protection was found only in UC patients who underwent appendectomy before the age of 20 years old [7]. Appendectomy performed at or prior to 20 years provides further protection in UC and CD patients [8]. However, similar results were not found in the present study. Hence, the effect of appendectomy age on CD onset needs to be further investigated in different races. The present study revealed that prior appendectomy was more frequent in Chinese CD patients and was a risk factor for the occurrence of CD. However, an increase in appendectomy rate may likely reflect a diagnostic bias.

The relationship between the appendectomy performed before CD diagnosis and the clinical course of CD was less and inconsistent. There are few relevant data in China. A study conducted in Australia revealed that appendectomy before CD diagnosis delayed the onset of disease but did not affect the disease severity [8]. However, another study conducted in Italy revealed a worsened clinical course of CD with increased risk of bowel resections in patients who underwent appendectomy before CD diagnosis [24]. A study conducted in France reported that prior appendectomy was associated with increased risk of stricture and a reduced risk of anal fistula but did not affect the severity of the disease [25]. The present study revealed no differences between patients who underwent appendectomy prior to CD diagnosis and patients who did not undergo this procedure in terms of clinical characteristics. When the appendectomy group was divided into subgroups by age of appendectomy (20 years), a statistical increase was shown for age at CD diagnosis in patients in the appendectomy group, who were appendectomized for over 20 years, when compared to the no appendectomy group. This result may mainly attribute to the fact that patients appendectomized for over 20 years when enrolled were older than those patients in the no appendectomy group. However, there is still lack of evidence in the present study to determine whether appendectomy may delay the onset of CD. These present results reveal that prior appendectomy does not influence the features or course of CD.

Due to the retrospective nature of the study, some of the data, such as the causes of appendectomy and present information about smoking, were not completely assessed. The present study only included inpatients from tertiary care hospitals, and selection bias was difficult to avoid. The number of patients and appendectomies were not sufficiently large. Hence, these results should be interpreted with these limitations.

In conclusion, this is the first intensive study that investigated the relationship between prior appendectomy and the onset and course of CD in Chinese patients. The rate of prior appendectomy increased in CD patients, and prior appendectomy was a risk factor for the onset of CD independent of smoking. However, this increased rate is only for the short duration before CD diagnosis, since as the duration was prolonged, this increase disappeared. This phenomenon suggests that prior appendectomy is unlikely associated with CD and likely reflects a diagnostic bias in the incipient CD. Furthermore, these present results reveal that prior appendectomy does not influence the phenotype or course of CD in Chinese patients.

## Figures and Tables

**Table 1:** The demographic characteristics of CD patients versus controls.

	CD (*n* = 617)	Controls (*n* = 617)	*P*
Age of CD diagnosis and control (median and range, years)	29 (6-79)	29 (6-78)	0.940
Male sex (%)	393 (63.70%)	393 (63.70%)	1.00
Han nationality (%)	602 (97.57%)	595 (96.43%)	0.243
Smokers (%)	219 (35.49%)	178 (28.85%)	0.012
Alcohol drinking (%)	103 (16.69%)	115 (18.64%)	0.370
NSAIDs use (%)	28 (4.54%)	22 (3.57%)	0.386

**Table 2:** Distribution of CD patients according to the Montreal classification.

CD (*n* = 617)			
Age at diagnosis (A)			
A1 (≤16 years)	54 (8.75%)		
A2 (17-40 years)	393 (63.70%)		
A3 (>40 years)	170 (27.55%)		
Location (L)		Upper GI modifier (L4)	
L1 (terminal ileum)	244 (39.55%)	L1+L4	12 (1.95%)
L2 (colon)	89 (14.42%)	L2+L4	1 (0.16%)
L3 (Ileocolon)	260 (42.14%)	L3+L4	11 (1.78%)
L4 (upper GI)	0		
Behavior (B)		Perianal disease modifier (p)	
B1 (nonstricturing, nonpenetrating)	189 (30.63%)	B1p	59 (9.56%)
B2 (stricturing)	211 (34.20%)	B2p	52 (8.43%)
B3 (penetrating)	95 (15.40%)	B3p	11 (1.78%)

**Table 3:** Appendectomy before CD diagnosis is stratified by CD location and behavior.

	Patients	Appendectomy (%)	Control (%)	*P* ^∗^
Location				
L1	256	21 (8.20%)	11 (4.30%)	0.099
L2	90	4 (4.44%)	3 (3.33%)	1.000
L3	271	16 (5.90%)	9 (3.32%)	0.230
Behavior				
B1	248	15 (6.05%)	8 (3.23%)	0.210
B2	263	17 (6.46%)	10 (3.80%)	0.248
B3	106	9 (8.49%)	5 (4.72%)	0.424
Bp	122	7 (5.74%)	5 (4.10%)	0.774
Total	617	41 (6.65%)	23 (3.73%)	0.033

^∗^Pairwise-matched analyses.

**Table 4:** The distribution of CD patients about age of appendectomy and interval between appendectomy and diagnosis of CD.

	Interval between appendectomy and diagnosis of CD (years)		
	0–1	2–5	>5
Age of appendectomy (year) in CD			
≤20	2	1	17
>20	4	6	11
Total	6	7	28
Age of appendectomy (year) in controls			
≤20	0	2	8
>20	0	0	13
Total	0	2	21

**Table 5:** Appendectomy versus no appendectomy in patients with CD.

	Previous appendectomy at or before 20 years (*n* = 20)		Previous appendectomy after 20 years (*n* = 21)		Total previous appendectomy patients (*n* = 41)		Nonprevious appendectomy patients (*n* = 576)
	Value	*P* ^∗^	Value	*P* ^∗^	Value	*P* ^∗^	Value
Age of CD diagnosis (median and range, years)	26 (15-52)	0.378	41 (24-70)	0.011	33 (15-70)	0.372	29 (6-79)
Male sex	14	0.586	10	0.124	24	0.477	369
Disease duration (month)	54.80 ± 40.46	0.823	51.76 ± 44.00	0.494	53.24 ± 41.81	0.441	47.93 ± 42.71
Smokers	5	0.322	8	0.827	13	0.600	206
Location of the disease		0.672		0.539		0.378	
L1	10		11		21		235
L2	2		2		4		86
L3	8		8		16		255
Behavior		0.452		0.545		0.691	
B1	9		6		15		233
B2	6		11		17		246
B3	5		4		9		97
Bp	2	0.398	5	0.573	7	0.653	115
Corticosteroids	7	0.386	9	0.861	16	0.473	258
Immunosuppressants	3	0.031	8	0.942	11	0.124	224
Anti-TNF agents	4	0.824	6	0.222	10	0.313	104
Intestinal resection	3	0.483	6	0.442	9	0.949	124

^∗^Compared with nonprevious appendectomy patients.

## Data Availability

The data used to support the findings of this study are available from the corresponding author upon request.

## Supplementary Materials

Supplementary Materials The data of the controls and the data of Crohn's disease patients.
